# Predicting pragmatic functions of Chinese echo questions using prosody: evidence from acoustic analysis and data modeling

**DOI:** 10.3389/fpsyg.2024.1322482

**Published:** 2024-02-27

**Authors:** Siyi Cao, Yizhong Xu, Tongquan Zhou, Anqi Wu

**Affiliations:** ^1^School of Foreign Languages, Southeast University, Nanjing, China; ^2^Department of Chinese Bilingual Studies, Hong Kong Polytechnic University, Kowloon, Hong Kong SAR, China; ^3^College of Foreign Languages, Nanjing University of Aeronautics and Astronautics, Nanjing, China

**Keywords:** Chinese echo questions, prosody, pragmatic functions, predicting models, machine learning

## Abstract

Echo questions serve two pragmatic functions (recapitulatory and explicatory) and are subdivided into two types (yes-no echo question and wh-echo question) in verbal communication. Yet to date, most relevant studies have been conducted in European languages like English and Spanish. It remains unknown whether the different functions of echo questions can be conveyed via prosody in spoken Chinese. Additionally, no comparison was made on the diversified algorithmic models in predicting functions by the prosodity of Chinese echo questions, a novel linguistic cognition in nature. This motivated us to use different acoustic cues to predict different pragmatic functions of Chinese echo questions by virtue of acoustic experiment and data modeling. The results showed that for yes-no echo question, explicatory function exhibited higher pitch and intensity patterns than recapitulatory function whereas for wh-echo question, recapitulatory function demonstrated higher pitch and intensity patterns than explicatory function. With regard to data modeling, the algorithm Support Vector Machine (SVM) relative to Random Forest (RF) and Logistic Regression (LR) performed better when predicting different functions using prosodic cues in both yes-no and wh-echo questions. This study from a digitized perspective adds evidence to the cognition of echo questions’ functions on a prosodic basis.

## 1 Introduction

Echo questions, a type of common and intriguing language phenomenon in verbal communication, involve partially or completely repeating the words of the previous speaker in an interrogative manner (Quirk, 2010). For example, in response to the preceding sentence “I planned a buy a cake as her present,” the listener can ask a wh-question “What did you buy?” since that he or she has not heard or understood the previous statement. Here, this kind of wh-question is the so-called echo question and it has two functions, i.e., explicatory and recapitulatory. The former function is to seek further clarification or elaboration, while the latter to elicit a repetition or confirmation of the preceding words (Quirk, 2010; Banfield, 2014). Due to the distinct functions, echo questions are commonly employed in specific scenarios, including hospitals (Zhang, 2018), interview programs (Oxburgh et al., 2010), and courts (Luo and Liao, 2012), effectively facilitating the communication between doctors and patients, interviewers and interviewees, judges and defendants, among others.

In addition to wh-question, echo questions have another form of question, i.e., yes-no question. For instance, the listener can also raise a yes-no question “You plan to buy a cake?” in order to seek the repetition of the speaker’s sentence “I planned a buy a cake as her present,” which serves as recapitulatory functions. However, there have been inconsistent voices on whether yes-no echo questions can serve explicatory functions. Quirk (2010) argued that wh-echo questions were linked with both recapitulatory and explicatory functions, but yes-no echo questions were only confined to recapitulatory functions. In contrast, in the view of Huddleson (1984), any question type (including yes-no echo questions) could fulfill explicatory functions. According to Blakemore (1994), yes-no echo questions could be used to meta-represent the preceding utterance or demand an account. Chen and Wen (2001) pointed out that the explicatory function occurs when listeners used an echo question to provide an explanation. On their account, whether yes-no echo questions served explicatory functions depended on listeners’ responses. This was likewise asserted by Leech and Short (2007) that listeners could utilize yes-no echo questions to seek further clarification when they could not comprehend the implicature of speakers. Evidently, these studies converge to indicate that yes-no echo questions can also serve explicatory functions when listeners offer an explanation. Accordingly, this study adopts the point that both yes-no echo questions and wh-echo questions can serve explicatory and recapitulatory functions. However, it remains unknown whether the different functions of echo questions can be conveyed via prosody.

### 1.1 Prosody of echo questions in different languages

Prosody serves as a key facilitator in aiding listeners’ comprehension of echo questions. In Germanic languages, particularly English, Hockey (1994) found that wh-echo phrases within wh-echo questions carried a high pitch accent (H*). However, Artstein (2002) proposed a rising pitch accent (L + H*) and a high-rising boundary (HH%) on focus in echo questions. German echo questions were examined by Repp and Rosin (2015), who found that those with a recapitulatory function have a higher intensity than those with an explicatory function. In Romance languages, such as Catalan, Prieto et al. (2015) noted different prosodic realizations of echo questions among dialects; for instance, Central and Northwestern Catalan used a rising intonation pattern, while Balearic Catalan opted for a falling one. Spanish wh-echo questions, as studied by Hualde and Prieto (2015), sought clarification through a circumflex ending or a low rise. Friulian echo questions were characterized by a L + ¡H* L% nuclear configuration due to declarative morphosyntax (Roseano et al., 2015). In Romance languages, the attention was primarily on explicatory functions in wh-echo questions and recapitulatory functions in yes-no echo questions, without scrutinizing the prosody of both functions for each question type. Clearly, there has not been a consensus on the prosodic realization of two distinct functions in both yes-no and wh-echo questions.

### 1.2 Prosody of echo questions in Chinese

Chinese echo questions availed themselves of specific features compared with echo questions of other languages, such as Germanic languages. First, Chinese is a tonal language, and tone plays a significant role in conveying meaning (Yip, 1980). Germanic languages, such as English or German, do not rely on tonal distinctions to the same extent (Liberman, 1975). Second, Chinese echo questions often maintain the same subject-verb-object (SVO) structure as the original statement or question, such as “你买了什么? (What did you buy?)” as wh-echo question and “你买了蛋糕? (You bought a cake?)” as yes-no question. In contrast, some Germanic languages, like English, might use typical questioning strategies and syntax, with wh-words usually appearing at the beginning of questions, such as “Who did Bill marry?” as wh pseudo echo questions (Parker and Pickeral, 1985). However, there has been a dearth of empirical studies on echo questions and their functions in Chinese linguistics.

To date, only three articles have been reported to specifically address echo questions from a prosodic perspective. Hu (2002) examined the prosodic expressions of interrogative words across three question types: wh-questions, yes-no questions and echo questions and found similar intonation patterns between wh-questions and recapitulatory echo questions. Luo and Liao (2012) claimed that the distinction between echo questions and declarative sentences could be unveiled by the use of boundary tone. Additionally, they observed that echo questions displayed higher f0 values compared with declarative sentences. Li et al. (2019) investigated the prosody of five pragmatic functions of yes-no echo questions and their corresponding statements, with the results showing that the overall F0 slope and average F0 can serve as indicators to differentiate between echo questions and statements. Yet, these three studies were problematic in the following six aspects: (1) lacking the investigation of prosody in explicatory echo questions; (2) leaving the prosody of pragmatic functions in wh-echo questions unexplored; (3) the five pragmatic functions can be consolidated into two functions; (4) failing to statistically report prosodic differences; (5) collecting small samples of data (e.g., 4 participants), far from enough to make a full statistical analysis; (6) exploring the prosody of echo question in a specified scene (e.g., courtroom discourse), leaving the issue unknown as to whether the result fits other contexts like daily communication. As a result, the prosodic realization in explicatory and recapitulatory functions in yes-no and wh-echo questions in spoken Chinese remains an open question.

### 1.3 Advanced modeling on predicting pragmatic functions using prosody

Modeling helps better clarify the intricate and multifaceted relationship between prosody and pragmatic functions. In most cases, prosody and pragmatic functions might not exhibit a linear relationship (Stolcke et al., 2000). Traditional models, such as ANOVA, assume linearity and struggle to capture the complex and non-linear mapping between prosody and pragmatics accurately (Lin, 2021). This challenge can be overcome with advanced machine learning (ML) techniques, known for their ability to decipher underlying relationships within data and handle complex linguistic problems, thus providing a more accurate predictive model, such as predicting different pragmatic functions using prosody (Lei and Liu, 2019).

In terms of speech recognition, constructing a model predicting speech recognition performance based on prosody using ML can significantly improve speech recognition systems’ ability to accurately recognize and interpret the prosody of different pragmatic functions (Amershi et al., 2014). This improvement contributes to enhancing the overall performance and user experience of human-machine dialogue systems. Furthermore, ML models can adapt to individual users’ speech patterns and continuously learn and make progress over time, ensuring personalized and precise interpretations of users’ prosody (Sacha et al., 2017).

### 1.4 Advanced modeling on Chinese echo questions

The current landscape of research within the Chinese language domain reveals a noticeable dearth of efforts directed toward the development of ML models for predictive analysis using acoustic cues. To date, only Shan (2021) validated this approach by creating a classification model of different pragmatic functions with the Random Forest technique, successfully predicting the functions of the Chinese discourse marker *nizhidao* based on its prosody. The reason why few studies developed predicting models of pragmatic functions using prosody based on advanced techniques is that a substantial number of computer professionals are engaged in the advancement of prosody recognition systems for speech processing (Vicsi and Szaszák, 2010), the majority tends to focus exclusively on the acoustic aspects without delving into the linguistic nuances, particularly those associated with pragmatics.

Echo questions in Chinese, characterized by their distinct prosody, play a pivotal role in pragmatic communication. Establishing a ML model for Chinese echo questions, particularly utilizing prosody to infer their pragmatic function, holds significant importance in theoretical and practical perspectives: theoretically, through a ML model, we can delve deeper into the linguistic features of Chinese echo questions, offering valuable insights for linguistic research, such as cross-linguistic comparative studies by exploring universal elements and language-specific variations in the expression of pragmatic functions through prosody. Moreover, traditional linguistic theories may not always explicitly consider prosody in pragmatic analyses. The learning process of the model can provide linguists with new perspectives, advancing linguistic theory. Practically, as mentioned before, one Chinese echo question has two different pragmatic functions and these functions are distinguished only by prosody. If the a computer or speech recognition system is unable to differentiate these two functions according to prosody, it may generate inaccurate or inappropriate responses, leading to decreased satisfaction with the technology and even a breakdown in the intended dialogue (Stolcke et al., 2000).

Backgrounded by the above advantages, this study attempts to construct a predicative model using ML techniques to further represent how Chinese echo questions employ prosody to convey meaning in verbal communication. In our study, we selected three advanced and representative models within the machine learning algorithms, namely Random Forest (RF), Support Vector Machine (SVM), and Logistic Regression (LR) for comparative evaluation. Each model was chosen for its unique strengths in addressing the specific characteristics of our problem. The RF, as a non-linear model, is exceptional in capturing complex relationships among the features (Degenhardt et al., 2019). SVM, particularly with an optimized kernel, demonstrates proficiency in navigating non-linear and high-dimensional spaces (Adam et al., 2014). In addition, LR provides a straightforward method for tracing linear relationships, while also offering probabilistic outputs (Maalouf, 2011). These models have been instrumental in past research, with ML algorithms like RF used in human-machine speech recognition to predict pragmatic functions based on prosodic parameters, overcoming the limitations faced by traditional models (Kim and Sohn, 2012). By deploying these three diverse, representative, and advanced ML algorithms, we aim to ensure a comprehensive exploration of potential patterns within pragmatics and prosody.

In a nutshell, the following two achievements were made on the previous researches regarding echo questions. First, an agreement has been roughly reached upon the recognition that both yes-no echo questions and wh-echo questions can serve explicatory and recapitulatory functions. Second, various pragmatic functions performed by distinct acoustic cues (including explicatory and recapitulatory functions) has been proved in many languages.

Despite the achievements obtained, two important issues are still unsettled as follows: (i) while various pragmatic functions performed by distinct prosodic cues have been demonstrated in German and other languages, their manifestations in spoken Chinese remain unexplored (e.g., Basnight-Brown and Altarriba, 2018); (ii) no predictive modeling has been made using prosodic cues in (i), although it is important in Chinese, particularly.

Backgrounded by the above, this study intends to explore the two following questions:

1. How are different pragmatic functions of echo questions conveyed by prosody in spoken Chinese?

2. Which machine learning model (Random Forest, Logistic Regression, Supported Vector Machine) is the most powerful in predicting the functions of echo questions in light of their prosodic patterns?

## 2 Method

### 2.1 Participants

In our study, 20 university students (10 males and 10 females) were recruited to participate in this experiment. All of them were native speakers of Mandarin. Besides, they spoke the Jianghuai dialect of Chinese, and had received the scores above Class B on the National Putonghua Proficiency Test (a national Chinese proficiency test for Chinese native speakers), indicating their proficiency in standard Chinese has not any discernible regional accent during daily communication. The age for all the participants ranged from 18 to 25 (*M* = 23.15, *SD* = 1.46). None of them had speech or hearing disorders according to their self-reports. Additionally, no participants had a history of mental illness or were diagnosed with psychiatric disorders. All participants demonstrated normal or corrected-to-normal vision. Self-reported data indicated that the majority were right-handed, as determined during the initial screening process.

This study referred to the experimental paradigm by Cao et al. (2019), receiving approval from the Human Research Ethics Committee from the university the first author affiliated to. Each participant was shown the experimental procedure clearly and signed the written informed consent prior to the experiment.

### 2.2 Materials

Nine pairs of target echo questions (i.e., wh-echo questions and yes-no echo questions) with a total of five words (S + V + N) were included as the materials, such as “你买了什么 (What did you buy?).” In order to control the variables of sentence length and syntactic position, all target sentences consisted of five words and the target focus was at the end of the sentence. In particular, the narrow focuses in target sentences were all interrogative pronouns (i.e., who, where, what) in wh-echo questions while proper nouns in yes-no echo questions concerned people, things and places. All the echo questions were revised from BCC corpus (Beijing Language & Culture University Corpus Center) (Xun et al., 2016) with the standard: (i) the sentences include echo questions; and (ii) the topic is related to the communication of people’s daily life. Given that each question type is associated with two functions, i.e., recapitulatory and explicatory, we produced four pairs of sentences as experimental stimuli: (1) wh-echo question with recapitulatory function, (2) wh-echo question with explicatory function, (3) yes-no echo question with recapitulatory function, (4) yes-no echo question with explicatory function. Specifically, given that Chinese echo questions with recapitulatory function results from the scenario in which a listener has not heard or understood a previous statement, the stimuli (1) and (3) provided two experiment conditions: one was set up in noisy environment (due to not having heard) and the other in the situation that the listeners have not understood the previous statement due to diverse reasons (e.g., a speaker’s unclear pronunciation). In order to elicit the target echo questions, the stimulating contexts were created in similar sentence patterns. That is why the stimulus “我买了他的最爱 (I bought his favorite thing)” was used to benefit speakers to understand the conversation and elicit the target explicatory echo questions “你买了什么? (What did you buy?)” (as shown in the following).

*@Wh-echo question*

*I. Recapitulatory function*

*Condition (1) (noisy environment):*

*(Hint): (环境嘈杂, 通话环境差, B在和 A 打电话, B没有听清 A说的买了什么)*

*(The environment is noisy, and the call quality is poor. B is on the phone with A and is unable to hear clearly what A said about buying)*

*A:今天小明生日, 我买了蛋糕,你看看还要买啥?*

*(Today is Xiaoming’s birthday. I bought a cake. You can see what you want to buy?)*

*B:你买了什么?*

*(What did you buy?)*

*A:买了蛋糕。.*

*(I bought a cake.)*

*Condition (2) (not understand):*

*(Hint): (A 和 B 边吃东西边聊天, B 有点口齿不清, A没有听清 B 想做什么)*

*(A and B are eating and chatting. B’s speech is a bit unclear, and A could not catch what B wants to do.)*

*A:周末咱们去哪玩好呢?*

*(Where should we go for the weekend?)*

*B:我看到有家 DIY 店挺不错的, 我想做蛋糕(发音不清).*

*(I saw a nice DIY shop; I want to make a cake (unclear pronunciation))*

*A:你想做什么?*

*(What do you want to do?)*

*B:蛋糕。.*

*(A cake.)*

*II. Explicatory function*

*(Hint): (B说买了小明最爱的东西, A想知道这个东西具体是什么)*

*(B said she bought something that Xiao Ming loves the most, and A wants to know specifically what this thing is)*

*A:今天小明生日, 送什么礼物好呢?*

*(Today is Xiaoming’s birthday. What should we buy?)*

*B:我买了他的最爱。.*

*(I bought his favorite thing)*

*A:你买了什么?*

*(What did you buy?)*

*B:他最喜欢的草莓蛋糕。.*

*(I bought his favorite strawberry cake)*

*@Yes-no echo question.*

*I. Recapitulatory function*

*Condition (3) (noisy environment):*

*(Hint): (环境嘈杂, 通话质量差, B在和 A 打电话, B 好像听到了蛋糕, 但不确定)*

*(The environment is noisy, and the call quality is poor. B are on the phone with A, and B seems to have mentioned something about cake, but it’s not certain)*

*A:今天小明生日, 我买了蛋糕,你看看还要买啥?*

*(Today is Xiaoming’s birthday. I bought a cake. You can see what you want to buy.)*

*B:你买了蛋糕?*

*(You bought a cake?)*

*A:对, 你不用再买蛋糕了。.*

*(Yes. You do not need to buy any other cakes.)*

*Condition (4) (not understand):*

*(Hint): (A 和 B 边吃东西边聊天, B 口齿不清, A 感觉好像听到了蛋糕, 但不确定,.*

*想确认一下).*

*(A and B are eating and chatting. B’s speech is unclear, and A feels like they heard “cake” but is not sure. A wants to confirm.)*

*A: 周末咱们去哪玩好呢?*

*(Where should we go for the weekend?)*

*B: 我看到有家 DIY 店挺不错的, 我想做蛋糕(发音不清).*

*(I saw a nice DIY shop; I want to make a cake (unclear pronunciation))*

*A:你想做蛋糕?*

*(You want to make a cake?)*

*B:对啊对啊。.*

*(Yes, exactly!)*

*II. Explicatory function*

*(Hint): (A 和 B 在讨论小明的生日礼物, 他们知道小明不喜欢吃奶油, 从来不吃蛋糕, A 想知道为什么 B 买蛋糕做礼物)*

*(A and B are discussing Xiao Ming’s birthday gift. They know Xiao Ming does not like cream and never eats cake. A wants to know why B is buying a cake as a gift)*

*A:今天小明生日, 送什么礼物好呢?*

*(Today is Xiaoming’s birthday. What should we buy?)*

*B:我买了蛋糕。.*

*(I bought a cake.)*

*A:你买了蛋糕?*

*(You bought a cake?)*

*B:我特地定做的无奶油的。.*

*(I bought a cake made especially without cream.)*

### 2.3 Procedure

Each participant was invited to the soundproof room to become familiar with the new environment and to complete individual reports, including name, gender, age and so on. Before the experiment, each participant was instructed to read and understand the provided hint in silence. Furthermore, they were specifically instructed not to portray the dialogues in a theatrical or overly dramatic fashion but instead to convey them vividly and consistently, reflecting their individual style. During the experiment, each participant was guided to play the role of either “B” or “A” depending on the text where the target sentence appears. For instance, if the target sentence was spoken by “A,” each participant would act as “A,” and the experimenter would then assume the role of “B” by reading the preceding sentence before the target sentence, thereby eliciting a natural response. The test began when the participants were ready and wore the headset microphone (2 inches away from the left side of their lips). During the process of recording, the participants were guided to read loudly and they would be stopped to read the sentences again when making any mistakes (e.g., overlooking target sentences). Moreover, both imaginary and real noise conditions may affect the prosody of echo questions (Scarborough et al., 2007). However, to date, no study has investigated potential differences between imaginary and real noisy environments. Therefore, in the experimental condition involving a noisy environment in this study, participants were initially instructed to imagine themselves in noisy surroundings, such as a bustling restaurant or a busy street. This setting required them to generate speeches in response to typical stimuli encountered in such environments. The recordings were all digitized in 44.1 kHz and 16-bit amplitude resolution and directly sampled and analyzed adopting the software of Praat.

### 2.4 Data analysis and modeling

Pitch, duration, and intensity are three main parameters adopted by experimental phonetics (Lehiste and Lass, 1976). Historically, research in this area has often depended on a limited set of experimental sentences, articulated by different participants to analyze sentence-level attributes such as intonation. Such a limited dataset might not effectively capture the acoustic intricacies of certain functions. To provide a more comprehensive analysis, we used various sentences with identical functions for our study. Following Pajupuu et al. (2015), we employed nine subdivided parameters of pitch, duration, and intensity, including f0Min, f0Max, f0Range, f0Mean, Duration, intensityMin, intensityMax, intensityRange, and intensityMean. These were extracted from target sentences spoken by native Mandarin Chinese speakers. In particular, the nine parameters were extracted from the syllables carrying narrow focus (i.e., the final two syllables, such as 什么 (what)).

All nine prosodic parameters were extracted from the ProsodyPro script (Xu, 2013) by virtue of the software Praat, a freely available software package for formatting and analyzing sound signals (Maryn, 2017). Specifically, four types of pitch values were examined and manually revised to correct pitch tracking errors. Simultaneously, these pitch values were extracted at 10 points, excluding voice cracks. To eliminate effects caused by gender differences (Cao et al., 2019), four parameters related to pitch (i.e., f0Min, f0Max, f0Range, f0Mean) were normalized and converted into *T*-values using the following formula (Shi, 1986):


$$T={\left(\left(\log\;\left(\mathrm{Target}\;\mathrm{Hz}\right)-\log\;\left(\min\right))/(\log\;\left(\max\right)-\log\;\left(\min\right)\right)\right)}^{*}\;5$$

A total of 720 sentences (4 functions*9 pairs*20 participants) were pooled in the present measurement from Chinese natives. The data of parameters of f0Min, f0Max, f0Range, f0Mean, Duration, intensityMin, intensityMax, intensityRange, intensityMean across two pragmatic functions in two types of echo questions were analyzed using R (R Core Team, 2016). A one-way analysis of variance (ANOVA) was adopted using EMMEANS function in “bruceR” package (Bao, 2023). Multiple comparisons using Tukey method were employed when a significant main effect was found.

Moreover, to construct a model that predicts pragmatic functions from specific prosodic parameters, and to accurately capture varying relationships within the data, we utilized three supervised learning algorithms: Random Forest (RF) (Qi, 2012), Support Vector Machine (SVM) (Karatzoglou et al., 2006), and Logistic Regression (LR) (Domínguez-Almendros et al., 2011).

Specifically, these three algorithms were compared to check which model could well predict different pragmatic functions in yes-no and wh-echo questions, i.e., to clarify which modeling results were more consistent with the data from the experiments themselves.

In the course of modeling, we referred to Jain et al. (2020) and adopted the statistical measures of accuracy, sensitivity (recall), specificity and F1 to evaluate the generalization capabilities of three models. For example, accuracy is simply a ratio of correctly predicted observations to the total observations.

Subsequently, to implement the three ML models in R, we utilized the randomForest function in “randomForest” package (RColorBrewer and Liaw, 2018) for RF algorithm, the svm function in “e1071” package for SVM modeling (Dimitriadou et al., 2006), and “glm” function in R to realize the LR algorithm (Turner and Firth, 2007).

## 3 Results

### 3.1 Prosodic pattern of yes-no echo question

**Figure 1:**
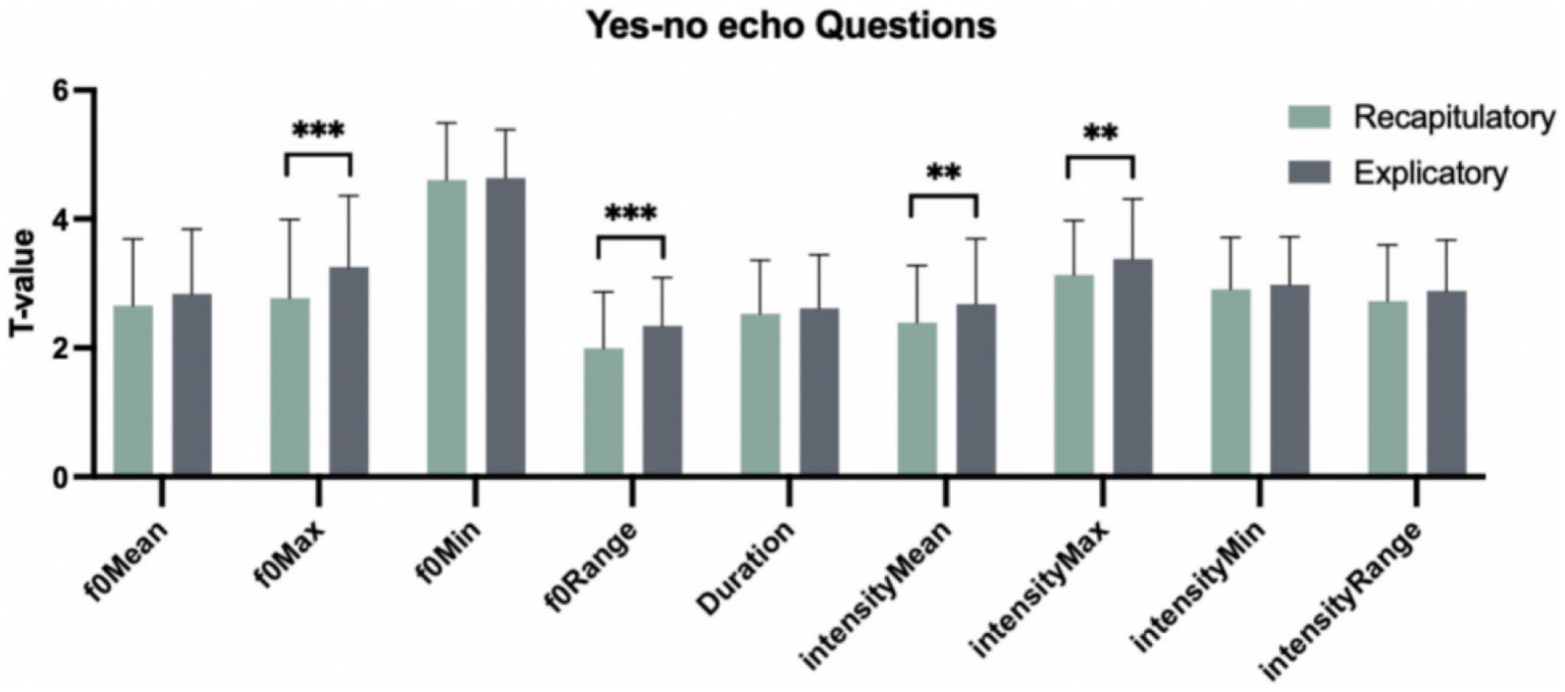
Nine prosodic parameters matching two functions in yes-no echo questions. *p < 0.05. **p < 0.01. ***p < 0.001.

Table 1 demonstrates the characteristics (i.e., *mean* and *SD*) of yes-no echo questions with recapitulatory and explicatory functions in terms of nine prosodic parameters, including f0Min, f0Max, f0Range, f0Mean, Duration, intensityMin, intensityMax, intensityRange, intensityMean. One-way analysis of variance (ANOVA) was conducted to test the different prosodic performances between two functions for yes-no echo question. Nine prosodic parameters were included as dependent variables and the factor of “Pragmatic function” (i.e., recapitulatory and explicatory function) as the independent variable. The results revealed the overall significant main effects of “Pragmatic function” in terms of the parameters f0Max, f0Range, intensityMean and intensityMax [*F*(1, 358) = 15.271, *p* < 0.001; *F*(1, 358) = 16.121, *p* < 0.001; *F*(1, 358) = 8.060, *p* < 0.01; *F*(1, 358) = 7.151, *p* < 0.01]. The multiple comparisons using Tukey method showed (Figure 1) that in yes-no echo questions, explicatory functions had the higher values than recapitulatory ones in terms of f0Max, f0Range, intensityMean and intensityMax [*β*(Explicatory–Recapitulatory) = 0.480, *t*(358) = 3.908, *p* < 0.001; *β*(Explicatory–Recapitulatory) = 0.346, *t*(358) = 4.015, *p* < 0.001; *β*(Explicatory–Recapitulatory) = 0.286, *t*(358) = 2.839, *p* < 0.01; *β*(Explicatory–Recapitulatory) = 0.250, *t*(358) = 2.674, *p* < 0.01]. This suggests that Chinese natives adopted the higher f0Max, f0Range, intensityMean and intensityMax to realize explicatory functions than recapitulatory functions in yes-no echo questions.

**Table 1:** Nine prosodic parameters matching recapitulatory and explicatory functions in yes-no echo questions.

	Recapitulatory		Explicatory				
Parameter	*M*	*SD*	*M*	*SD*	*F(1, 358)*	*p*	*ηp^2^*
f0Mean	2.654	1.038	2.837	1.005	2.889	0.090	0.008
f0Max	2.771	1.221	3.252	1.108	15.271	<0.001	0.041
f0Min	4.604	0.886	4.636	0.752	0.139	0.709	0.000
f0Range	1.992	0.877	2.338	0.754	16.121	<0.001	0.043
Duration	2.527	0.832	2.614	0.832	0.974	0.324	0.003
intenistyMean	2.391	0.888	2.678	1.019	8.060	0.005	0.022
intensityMax	3.132	0.842	3.382	0.928	7.151	0.008	0.020
intenistyMin	2.910	0.807	2.982	0.747	0.770	0.381	0.002
intenistyRange	2.726	0.875	2.883	0.794	3.183	0.075	0.009

### 3.2 Prosodic pattern of wh-echo question

**Figure 2:**
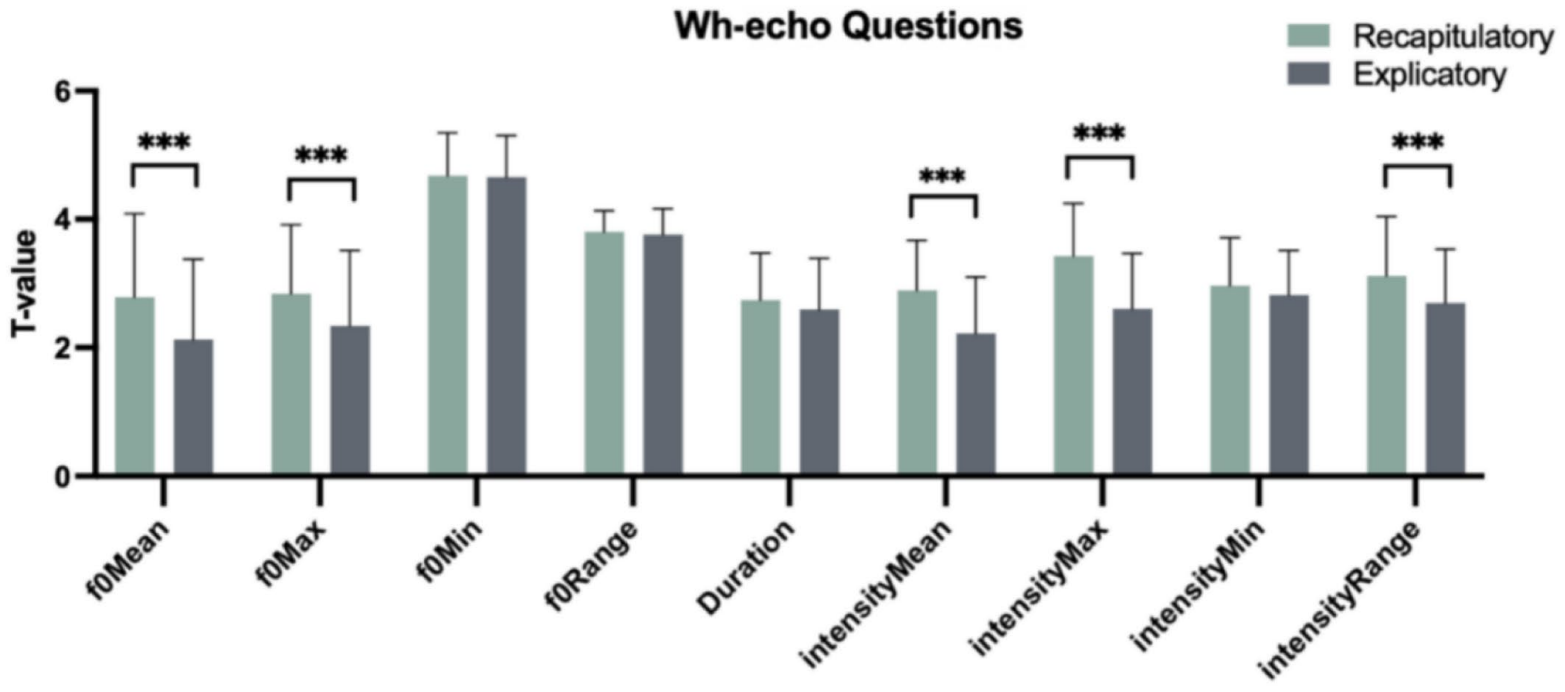
Nine prosodic parameters matching two functions in wh-echo questions. *p < 0.05. **p < 0.01. ***p < 0.001.

Table 2 displays the features of wh-echo questions with recapitulatory and explicatory functions in light of the same prosodic parameters as mentioned above. One-way analysis of variance (ANOVA) analysis revealed a statistically significant difference between these two pragmatic functions. Specifically, there were significant main effects of “Pragmatic function” with respect to the parameters of f0Mean, f0Max, intensityMean, intensityMax and intensityRange [*F*(1, 358) = 23.945, *p* < 0.001; *F*(1, 358) = 17.336, *p* < 0.001; *F*(1, 358) = 58.152, *p* < 0.001; *F*(1, 358) = 86.882, *p* < 0.001; *F*(1, 358) = 20.597, *p* < 0.001]. The multiple comparisons using Tukey method showed (Figure 2) that in wh-echo questions, explicatory functions transmitted lower values than recapitulatory functions in terms of f0Mean, f0Max, intensityMean, intensityMax and intensityRange [*β*(Explicatory–Recapitulatory) = −0.659, *t*(358) = −4.893, *p* < 0.001; *β*(Explicatory–Recapitulatory) = −0.494, *t*(358) = −4.164, *p* < 0.001; *β*(Explicatory–Recapitulatory) = −0.667, *t*(358) = −7.626, *p* < 0.001; *β*(Explicatory–Recapitulatory) = −0.825, *t*(358) = −9.321, *p* < 0.001; *β*(Explicatory–Recapitulatory) = −0.423, *t*(358) = −4.538, *p* < 0.001]. This implies that Chinese natives used the lower f0Mean, f0Max, intensityMean, intensityMax and intensityRange to realize explicatory than recapitulatory functions in wh-echo questions.

**Table 2:** Nine prosodic parameters matching recapitulatory and explicatory function in wh-echo questions.

	Recapitulatory		Explicatory				
Parameter	*M*	*SD*	*M*	*SD*	*F(1, 358)*	*p*	*ηp^2^*
f0Mean	2.784	1.303	2.126	1.251	23.945	<0.001	0.063
f0Max	2.835	1.080	2.341	1.170	17.336	<0.001	0.046
f0Min	4.677	0.670	4.656	0.645	0.091	0.763	0.000
f0Range	3.804	0.331	3.743	0.488	1.939	0.165	0.005
Duration	2.740	0.732	2.592	0.802	3.358	0.068	0.009
intenistyMean	2.892	0.778	2.225	0.878	58.152	<0.001	0.140
intensityMax	3.428	0.815	2.603	0.863	86.882	<0.001	0.195
intenistyMin	2.962	0.749	2.817	0.696	3.623	0.058	0.010
intenistyRange	3.116	0.926	2.693	0.841	20.597	<0.001	0.054

### 3.3 Data modeling of yes-no echo questions

According to the results in 3.1, the four parameters, i.e., f0Max, f0Range, intensityMean and intensityMax were adopted to predicting different pragmatic functions using prosodic cues. The results using 10-fold cross-validation in Table 3 showed that SVM produced the highest overall classification accuracy (83%) among LR (75%) and RF (69%), suggesting that SVM represents the highest probability that the randomly selected sound was classified correctly as the pragmatic function in yes-no echo question. Furthermore, SVM demonstrated the best performance among three models with 87.5 sensitivity, 80% specificity and 82% F1. Although the model of LR performed better than RF in sensitivity (80% vs. 66%) and F1 (72% vs. 71%), the classification specificity of LR was lower than RF (72% vs. 74%). In one word, SVM performed better than other two models to predict pragmatic functions using prosodic cues in yes-no echo question.

**Table 3:** Accuracy, sensitivity, specificity and F1 of different methods in yes-no echo questions.

**Method**	**Accuracy (%)**	**Sensitivity (%)**	**Specificity (%)**	**F1 (%)**
SVM	83	87.5	80	82
LR	75	80	72	72
RF	69	66	74	71

### 3.4 Data modeling of wh-echo questions

Based on the results of 3.2, five prosodic parameters (i.e., f0Mean, f0Max, intensityMean, intensityMax and intensityRange) were adopted to construct the model predicting pragmatic functions using prosodic cues. In light of classification accuracy, sensitivity, specificity and F1, the results using 10-fold cross-validation in Table 4 showed that SVM had the overall higher classification accuracy (86%) than LR (66%) and RF (77%), implying that SVM indicates the highest probability that the classifier has correctly classified the randomly selected sounds into the pragmatic function in wh-echo questions. What’s more, in contrast with the data in yes-no questions, RF performed better than LR in overall classification sensitivity (73% vs. 69%), specificity (86% vs. 65%) and F1 (79% vs. 64%). To conclude, SVM performed better than other two models in distinguishing the two functions in wh-echo questions.

**Table 4:** Accuracy, sensitivity, specificity and F1 of the proposed methods in wh-echo questions.

**Method**	**Accuracy (%)**	**Sensitivity (%)**	**Specificity (%)**	**F1 (%)**
SVM	86	81	93	87
LR	66	69	65	64
RF	77	73	86	79

## 4 Discussion

The current study resorted to an acoustic experiment to explore whether the different pragmatic functions (i.e., recapitulatory and explicatory) of two types of echo question types (i.e., yes-no echo question and wh-echo question) can be conveyed via prosody and modeled in spoken Chinese. That is, whether different pragmatic functions of echo question can be realized prosodically and whether a classification model can be constructed to predict functions using prosodic cues. The results are summarized from two folds as below.

Firstly, Chinese natives did employ different prosodic cues to realize various pragmatic functions. Secondly, the comparison of three machine learning algorithms indicated that SVM performed better than RF and LR when predicting pragmatic functions using prosodic cues in two echo question types. This suggests that different pragmatic functions can be revealed and understood by its prosody. What follows elaborates on the possible factors to motivate the results.

### 4.1 Prosodic variations of different pragmatic functions in yes-no echo questions

For the yes-no echo question type, explicatory questions exhibited higher pitch pattern (i.e., f0Max, f0Range) and intensity pattern (i.e., intensityMean and intensityMax) than recapitulatory questions in our experiment. This result reveals that yes-no questions can act as explicatory echo functions as well, which is inconsistent with previous studies (Quirk, 2010).

Our statistical data do not align with the theoretical view that yes-no questions cannot serve explicatory functions. According to Quirk (2010), explicatory functions within wh-echo questions, specifically those involving wh-words like “what” and “who,” can only be fulfilled by wh-echo questions. These questions are employed to directly elicit clarification from listeners. Nevertheless, Quirk (2010) ignored the effect of context which can allow yes-no echo question to deliver explicatory implications. For example, imagine that two interlocutors are talking about Mary’s birthday and Mary is lactose intolerant, the listener is confused with the response of “I plan to buy a cake as her present” and raises a yes-no question “Do you plan to buy a cake?” At this moment, this yes-no question is employed to ask for a reason rather than to seek a repetition. Evidently, a yes-no question is able to be shifted into an explicatory echo question under certain contexts.

The results reveal that the pitch values of f0Max were higher in explicatory echo questions than in recapitulatory echo questions. This discrepancy is believed to be driven by the degree of confidence the speaker wishes to convey to his utterances. According to Braga and Marques (2004), the pitch rise or fall correlates with the speaker’s confidence level in their utterances. In simpler terms, the lower degree of confidence results in increased pitch rise, as evidenced by higher f0Max. In our test, explicatory echo questions were posed to seek clarification, indicating that the listener was doubtful why the speaker made the preceding statement, which might conflict with their own understanding. This suggests that the listener lacks confidence in the content and seeks further elucidation or feedback from the speaker. In contrast, recapitulatory echo questions were intended to confirm understanding, signaling that the listener grasped the speaker’s meaning but remained uncertain. This implies that the listener is more confident in the content but still seeks confirmation. Consequently, the pitch values of f0Max in explicatory echo questions were higher than those in recapitulatory echo questions.

Additionally, the findings indicate that the pitch values of f0Range were higher in explicatory echo questions than in recapitulatory echo questions. f0Range is calculated as the difference between f0Max and f0Min in intonation. This phenomenon is believed to be influenced by two factors. Firstly, Chinese, like many languages, utilizes intonation to convey meaning and subtlety (Kratochvil, 1998). Explicatory echo questions may adopt a rising intonation pattern that denotes curiosity, surprise, or emphasis, resulting in higher pitch values for f0Range. Conversely, recapitulatory echo questions may exhibit a more stable or falling intonation pattern associated with affirmation or confirmation, leading to lower f0Range. Secondly, according to Braga and Marques (2004), the pitch range increases when new information segments of speech emerge. In our study, the target word at the end of yes-no echo questions, such as “蛋糕 (cake)” in the phrase “你买了蛋糕? (You bought a cake?),” carries new or focal information with explicatory function to request clarification. By increasing the f0Range, the speaker intends to evoke listeners’ attention and get more information or feedback from the speaker.

In addition to pitch values, explicatory echo questions had higher intensity values than recapitulatory echo questions. This result is perhaps attributed to a positively correlation between f0 and intensity (Vaissière, 1983). In other words, when F0 increases or decreases, intensity tends to increase or decrease concurrently (Vaissière, 1983; Shan, 2021). In fact, the correlation between intensity and F0 is based on physiological principles (Wu, 2002), which are controlled by the same mechanical processing, i.e., tension in the vocal folds or increase in sub-glottal pressure (Vaissière, 1983). In this regard, when the f0 values of explicatory echo questions are higher than that of recapitulatory questions, the intensity demonstrates the same phenomenon accordingly.

What’s more, intensity is generally related to the emotional states of speakers (Juslin and Laukka, 2001). The majority of explicatory echo questions contain a strong feeling of doubts while recapitulatory echo questions express the emotion of uncertainty. According to Rilliard et al. (2013), the emotion of doubts (normalized: *M* = 0.84) has the higher intensity than that of uncertainty (normalized: *M* = −0.53), explaining why the intensity values of explicatory echo questions were higher than those of recapitulatory echo functions in this study.

### 4.2 Prosodic variations of different pragmatic functions in wh-echo questions

The experiment shows that explicatory echo questions had the lower pitch (i.e., f0Mean, f0Max) and intensity values (i.e., intensityMean, intensityMax, and intensityRange) than recapitulatory echo questions. Several factors may account for these results. Firstly, high-pitched recapitulatory functions in wh-echo questions may result from speakers’ more effort to convey the recapitulatory meanings. Duffley and Enns (1996) claimed that wh-words (i.e., what, where, etc.) are more often than not used to ask for a clarification of the thing or the place, suggesting that wh-echo questions *per se* avail themselves of explicatory function and speakers do not need to spend more energy to deliver such function. Hence, in order to seek a repetition or confirmation via wh-echo questions, speakers have to place more stress on the sentence, such as increasing F0 values. In our experiment, participants were required to use wh-echo questions to express recapitulatory functions, thus they spent more efforts by raising the pitch values to utter the question.

Secondly, the high f0 of recapitulatory functions is supposed to be more correlated with talking in a noisy environment. In this study, in order to elicit recapitulatory functions in wh-echo questions, one of the stimuli was intentionally placed in a noisy setting. Specifically, the participants were instructed to imagine that they were communicating in a noisy environment, which design referred to Scarborough et al. (2007): stressed vowels were longer, speech rate was slower, and vowel space distances were expanded in imagined scenarios of foreigner-directed speech (FDS) compared to real FDS scenarios. It seems that imagined FDS tends to manifest more pronounced prosodic variations than its real counterpart. Notably, there has been no exploration of potential disparities in Lombard speech, namely the speech style in noisy environments (Lombard, 1911; Tang et al., 2017), between imagined and real noisy settings. In contrast, actual noisy environments prompt Lombard speech to adopt a higher pitch, enhancing phonetic contrast relative to speech produced in quiet surroundings (refer to Junqua, 1996, for a comprehensive review). Assuming a similar trend in imagined Lombard speech, mirroring the pattern observed in imagined FDS, this might elucidate why pitch values were higher in recapitulatory echo questions compared to explicatory echo questions.

Thirdly, high intensity values were observed concurrently in recapitulatory functions in the wh-echo questions. Repp and Rosin (2015) revealed that in German, wh-words in echo questions signaling auditory failure had higher intensity than those in echo question with information-seeking. Yet, it was reported that in French, echo questions expressing auditory failure in wh-echo questions were not marked by higher intensity. Our results were consistent with Repp and Rosin (2015), but inconsistent with Glasbergen-Plas et al. (2021), indicating that echo questions may display divergent prosodic features in different languages even though the questions are related to the same pragmatic functions.

Our findings show that for yes-no echo question, explicatory function exhibited higher pitch and intensity patterns than recapitulatory function. In contrast, for wh-echo question, recapitulatory function demonstrated higher pitch and intensity patterns than explicatory function. The potential difference in prosodic performance between wh-echo and yes-no echo questions can be attributed to the nature of the final two syllables. In our study, wh-echo questions conclude with interrogative words such as什么 “what” at the end of the sentence, inherently carrying a questioning tone due to their interrogative nature. This characteristic might lead to a tendency for higher pitch in recapitulatory wh-echo questions, reinforcing the interrogative nature of the final syllable. Conversely, yes-no echo questions end with noun words such as 蛋糕 “cake,” which are declarative in nature and lack the inherent questioning tone of interrogative words. Therefore, recapitulatory yes-no echo questions might require a higher pitch to emphasize the explicatory function, which seems to emulate the question-like quality of the statement.

Additionally, our study is limited by the fact that we did not consider the tone of the final two syllables in the two different types of echo questions. This limitation arises from the restricted availability of wh-words in Mandarin Chinese. To control for the influence of the position of the target word on the results on both echo questions, we placed the target wh-words at the end of sentences, and only three wh-words (i.e., what, where, who) could be positioned in this manner. More future studies are desired to explore whether the tone of Mandarin Chinese can affect the realization of different pragmatic functions through prosody in echo questions.

### 4.3 SVM performing better than RF and LR in predicting functions using prosodic cues

The modeling results illustrate that SVM provides the classification performance advantages compared with RF and LR. This is believed to result from the fact that the acoustic data were collected from real but limited participants, which means that the size of participants is relatively small and all the data is non-linear (i.e., there is not a direct and clear relationship between variables). SVM is a versatile classification algorithm models constructed on the basis of small data instances from different classes (Yu et al., 2012). Besides, it can provide another efficient method: a nonlinear kernel function so as to fit the nonlinear data (Adam et al., 2014). Therefore, the classification error is considerably minimized.

Compared with SVM, RF may fail to obtain good results from small data. RF combines tree classifiers so that each tree classifier depending on randomly independent samples in RF spits out a class prediction and the class with the most votes becomes the model’s prediction (Kim and Sohn, 2012). Nevertheless, RF is not good for small data or low-dimensional data (data with few features) (Degenhardt et al., 2019). The data in this study are composed of only nine features (i.e., four in yes-no echo questions and five features in wh-echo questions), far from enough to construct a good RF model.

Likewise, LR cannot solve the non-linear problems. LR builds a regression model to predict the probability that a given datum entry belongs to the specific category (Maalouf, 2011). However, non-linear data cannot be well fitted using LR since this model has a linear decision surface, for linearly separable data are rarely found in real-world scenarios. In this study, the collected acoustic data of echo questions with four or five parameters are all non-linear in nature. That is why LR model performed the worst among all the models.

However, one notable limitation of aforementioned ML models lies in the potential influence of multicollinearity among prosodic parameters, which is caused by the interrelated nature of prosodic parameters, such as f0Max and f0Range. Our upcoming study may consider to adopt advanced statistical techniques such as ridge regression or principal component analysis so as to mitigate multicollinearity among prosodic parameters, which may offer robust approaches to handle multicollinearity and enhance the reliability of further ML predictive models.

## 5 Conclusion

This study employed a phonetic experiment to display how different pragmatic functions (recapitulatory and explicatory functions) was conveyed via prosody in uttering Chinese echo questions (yes-no question and wh-question). The results show that explicatory functions display higher pitch and intensity values than recapitulatory functions in yes-no echo questions, but recapitulatory functions demonstrate higher pitch and intensity values than explicatory functions in wh-echo questions. By using the experimental data, three machine learning algorithms (i.e., Random Forest (RF), Logistic Regression (LR), Supported Vector Machine (SVM)) were compared, in predicting functions using acoustic cues, with results showing that SVM performed better than RF and LR in predicting pragmatic functions using prosodic cues both in yes-no and wh-echo questions.

Our study extends the previous studies in that experiment-based speech analyses and computational statistics can be well combined in echo question research. The statistical model clearly and validly duplicates both the abstract pragmatic functions of echo question and the intrinsic mechanism of predicting models, as found in the acoustic experiment.

The challenge for future research is to explore the prosody of specific emotions conveyed via echo question and combine machine learning with traditional speech research to benefit speech recognition, such as have the computer automatically recognize the specified population based on prosodic features.

## Data availability statement

The raw data supporting the conclusions of this article will be made available by the authors, without undue reservation.

## Ethics statement

The studies involving humans were approved by Nanjing University of Aeronautics and Astronautics. The studies were conducted in accordance with the local legislation and institutional requirements. Written informed consent for participation in this study was provided by the participants’ legal guardians/next of kin.

## Author contributions

SC: Writing – original draft, Writing – review & editing. YX: Conceptualization, Writing – original draft, Writing – review & editing. TZ: Writing – review & editing. AW: Data curation, Methodology, Writing – review & editing.
